# Analysis of Additive Distributions in Rigid PVC by Laser Ablation Inductively Coupled Plasma Mass Spectrometry

**DOI:** 10.3390/polym18212626

**Published:** 2026-10-23

**Authors:** Saskia Kerkeling, Martin Kreyenschmidt, Christian Gieler, Matthias Kaltefleiter-Jürgens, Michel Sieffert, Ursula E. A. Fittschen

**Affiliations:** 1 Department of Chemical Engineering, FH Münster University of Applied Sciences, 48565 Steinfurt, Germany; 2 VEKA AG, 48324 Sendenhorst, Germany; cgieler@veka.com (C.G.);; 3 Gealan Fenster-Systeme GmbH, 95145 Oberkotzau, Germany; msieffert@gealan.de; 4 Institute of Inorganic and Analytical Chemistry, Clausthal University of Technology, 38678 Clausthal, Germany; ursula.fittschen@tu-clausthal.de

**Keywords:** PVC, Laser ablation, ICP-MS, spatially resolved analysis, additive migration, window profiles, artificial weathering

## Abstract

Laser ablation inductively coupled plasma mass spectrometry (LA-ICP-MS) enables direct, spatially resolved analysis of inorganic additives and impurities in rigid PVC, which is widely used in window profiles with demanding lifetime and recycling requirements. This study develops and validates a matrix-adapted LA-ICP-MS protocol for depth-resolved profiling of key additive elements (Ca, Mg, Al, Ti, Zn) and trace Fe in PVC window profiles and laboratory specimens. Laser fluence was optimized, and instrumental precision, positional variability, and ablation depth increments were quantified to ensure reproducible profiles over the first ~25 µm. Subsequently, the method was applied to pressed sheets, extruded belts, and window profiles. The last included samples produced under varying calibration vacuum and those subjected to xenon arc UV/water exposure. Extruded profiles exhibited denser, Zn- and TiO_2_-enriched, Fe-contaminated skin layers compared with laboratory specimens, while increasing calibration vacuum enhanced near-surface enrichment of several elements. Artificial weathering led to pronounced depth-resolved depletion of Ca, Mg, and Zn in the near-surface region, consistent with stabilizer consumption and leaching of soluble chlorides, whereas Al and Ti remained essentially constant. These results demonstrate that processing strongly shapes near-surface additive and impurity distribution in PVC profiles and that LA-ICP-MS provides a powerful tool to link depth-resolved inorganic composition to aging mechanisms and to assess the transferability of laboratory weathering results to real window profiles.

## 1. Introduction

Rigid PVC is a predominant thermoplastic in the construction sector [1,2], particularly for window profiles. The requirement for extended service lifetimes, coupled with rising recycling rates [3], necessitates exceptional aging stability. Industrial PVC formulations for profiles typically contain complex additive packages, including calcium/zinc-based stabilizers, magnesium and aluminum-containing co-stabilizers, inorganic fillers such as calcium carbonate, and pigments such as titanium dioxide. These components introduce elements such as Ca, Zn, Mg, Al, Ti, and trace Fe into the materials and strongly influence processing behavior, weather resistance, and degradation performance [4,5,6].

Numerous studies have focused on elucidating the molecular degradation mechanisms of PVC, including HCl elimination and polyene formation analyzed by Raman [7,8,9] or UV/Vis spectroscopy and oxidative processes characterized by infrared spectroscopy (IR) [10,11,12,13,14]. However, these techniques primarily probe changes in the polymer backbone and do not provide spatially resolved information on the distribution of inorganic additives and impurities.

In practice, the spatial distribution of these additives and impurities is shaped both by processing and by subsequent weathering. During extrusion and calibration, migration processes can occur. This is desirable for external lubricants, but such mobile components can also act as carriers for inorganic additives [15]. Moreover, processing steps inevitably introduce local contaminations, particularly at surfaces in contact with equipment and cooling media. During service, weathering processes such as UV irradiation, temperature cycling, and water exposure likewise act predominantly at the surface [16] and can induce local depletion or enrichment of stabilizers and other inorganic species [17]. As a result, the local availability of additives at or near the surface may differ substantially from the bulk composition. To understand such processing-induced inhomogeneities and the effects of artificial or natural aging, it is therefore essential to obtain spatially resolved, depth-dependent information on the elemental distribution in rigid PVC.

Laser ablation inductively coupled plasma mass spectrometry enables direct analysis of inorganic elements in solid polymers with minimal sample preparation and provides spatially resolved information when combined with time-resolved detection and controlled sample movement [18,19,20]. For rigid PVC, LA-ICP-MS can measure not only key additive elements (Mg, Al, Ca, Ti, Zn), but also trace metal impurities, including transition metals such as Cr, Mn, Fe, Co, and Cu, which are known to catalyze polymer degradation *via* the hydroperoxide decomposition pathway [16]. By recording successive ablations at the same position, LA-ICP-MS can generate depth profiles that resolve variations in elemental concentration with depth, including near-surface enrichment caused by migration processes as well as depletion or leaching effects resulting from weathering [21,22]. Although LA-ICP-MS has been applied to other industrial polymers [23,24], matrix-adapted, depth-resolved analysis of PVC window profiles and aging effects has not yet been systematically explored.

Depth-resolved information on additive distributions is particularly important for understanding the mechanisms of aging in PVC window profiles. Photochemical weathering primarily affects a thin near-surface region [25] where gradients in stabilizers, pigments, and impurities can strongly influence UV interaction and degradation rates [26,27]. When profiles are evaluated for service life or collected as recyclate, changes in additive distributions and migrated species at the surface can impact both their performance and their behavior during reprocessing. A characterization technique capable of resolving elemental depth profiles before and after artificial weathering is therefore valuable for linking local additive state to degradation behavior and for assessing the impact of weathering on PVC window profiles.

In industrial practice, aging behavior and formulation changes are often evaluated using laboratory specimens such as rolled and pressed sheets or extruded belts [4,12,28], which can be produced quickly from limited amounts of raw material. The results obtained on these samples are then extrapolated to window profiles. However, differences in processing routes between laboratory specimens and industrial profiles mean that the properties of the test specimens, such as their internal structures and additive states, may not fully reflect those of the end products. A systematic comparison of additive depth profiles in these specimen types is therefore required to assess the validity of laboratory aging tests and the transferability of their results to real products.

The aim of this work is to develop and validate a matrix-adapted LA-ICP-MS method for rigid PVC that enables reproducible, depth-resolved analysis of inorganic additives and impurities in the near-surface region. The method is first optimized (laser fluence) and statistically validated (system precision, positional variability) for PVC window profiles. It is then applied to compare depth-resolved elemental distributions in specimen types commonly used in aging studies (pressed sheets, extruded belts, and industrial window profiles) and investigate changes in additive depth profiles induced by artificial aging in a xenon arc weathering tester. By linking processing-induced and aging-induced variations in stabilizer, pigment, and impurity distributions, particularly surface enrichment of zinc stearate, TiO_2_, and Fe, to the underlying degradation mechanisms, the study provides a basis for understanding PVC aging behavior and assessing the suitability of different specimen types for accelerated weathering tests and recycling evaluations.

## 2. Materials and Methods

### 2.1. Instrumentation and Operating Conditions

Laser ablation (LA) was performed using an Analyte Excite 193 nm ArF excimer laser equipped with a HelEx II tunable 2-volume ablation cell (Teledyne Photon Machines, MT, USA) under the conditions summarized in Table 1. The ablation cell was operated with two helium flows: one introduced into the outer cell (He1) and a second introduced into the inner cell (He2), positioned directly above the sample in the ablation region to optimize aerosol removal.

The LA system was coupled to an 8900 Triple Quadrupole ICP-MS (Agilent Technologies, Japan), operated under the conditions summarized in Table 2. Ablated material was transported from the ablation cell to the ICP-MS plasma by a constant helium carrier gas flow. Upstream of the plasma torch, a gas-mixing bulb was installed to combine the helium aerosol stream with the argon nebulizer gas, ensuring efficient aerosol transport and stable plasma conditions. To minimize polyatomic interferences, the ICP-MS was operated in MS/MS mode with a collision cell using helium as collision gas. Instrumental parameters were tuned regularly using NIST SRM 612 glass to achieve maximum sensitivity (monitored *via* ^89^Y^+^, ^139^La^+^, and ^238^U^+^), low oxide formation (evaluated as the ^248^ThO^+^/^232^Th^+^ ratio), and minimal elemental fractionation (evaluated as the ^232^Th^+^/^238^U^+^ ratio).

### 2.2. Sample Production

All samples investigated in this study were produced and supplied by VEKA AG (Germany) based on a standard PVC formulation for window profiles. The formulation contained PVC, an impact modifier, a processing aid, internal and external lubricants, calcium carbonate (chalk), titanium dioxide, and a commercial One Pack Ca/Zn stabilizer for PVC window profiles. As this stabilizer is a commercial One Pack product, its complete proprietary formulation is not disclosed by the manufacturer. However, such stabilizer systems typically contain calcium and zinc stearates or hydroxy stearates, magnesium hydroxide, hydrotalcite, and additional co-stabilizers. The detailed composition and material characteristics are given in Supplementary Table S1. A dry-blend powder mixture was prepared from the raw materials on an industrial scale and used to produce pressed sheets, extruded belts, and window profiles. The approximate mass fractions of the key elements (Ca, Mg, Al, Ti, Zn) were determined via wet chemical analysis, with detailed methodological parameters and results reported in [29]. These values, which serve as the baseline for the LA-ICP-MS depth profiling, are summarized in Table 3.

Pressed sheets were prepared using a two-roll mill followed by pressing in a hydraulic press. Extruded belts were produced with a single-screw extruder equipped with sheet dies and a three-roll sheet take-off. Window profiles were manufactured using two different twin-screw extruders operated in mono- and co-extrusion modes with die geometries representative of industrial production. These differences in extruder type, extrusion mode, and die design result in distinct processing conditions, particularly with respect to melt temperature, melt pressure, and extrusion speed, which in turn can influence the distribution of additives in the PVC matrix.

In industrial practice, laboratory samples such as pressed sheets and extruded belts are frequently used for aging studies and to investigate the effects of formulation changes, because they can be produced quickly from relatively small amounts of raw material and independently of routine production. Extruded window profiles, by contrast, most closely represent real products but require larger raw material quantities and available production capacity. To assess the suitability of these different specimen types for aging studies and the transferability of laboratory results to real profiles, their elemental compositions and associated additive depth distributions were compared in this work.

To investigate the influence of post-extrusion calibration on the elemental distribution within window profiles, additional profiles were produced with varying vacuum levels in the calibration unit. A vacuum of 100% corresponds to the negative pressure required to obtain a marketable product under standard processing conditions (−0.3 bar). In subsequent tests, this negative pressure was reduced to 50%, and in a further variant, no vacuum (0%) was applied during calibration. These profiles were analyzed by LA-ICP-MS depth profiling to assess whether the calibration vacuum affects additive distributions in the near-surface region and bulk.

Further details on the processing equipment and specific operating parameters used for the preparation of the test specimen are provided in the Supplementary Materials (Tables S2–S5).

### 2.3. Fluence Optimization and Validation of Depth-Profiling

To adapt the laser ablation conditions to the PVC matrix, the laser fluence was optimized with respect to signal intensity and precision, expressed as relative standard deviation (RSD), for Mg, Al, Ca, Ti, and Zn. Fluence values between 1 and 5 J cm^−2^ were investigated. For each fluence setting, three replicate line ablations of 2.5 mm length were performed on a PVC window profile.

To validate the feasibility of systematically analyzing PVC window profiles by LA-ICP-MS and generating reproducible depth profiles, the influence of the measurement system and the sampling position was investigated using industrially manufactured window profiles. Depth profiles were obtained by performing five consecutive ablations at identical x, y, and z coordinates along a 1 cm line, thereby removing successive layers of material. To assess instrument stability (system precision), this depth-profiling procedure was repeated five times, with the spacing between the lines kept as small as possible to minimize the effect of sample inhomogeneity. In a second experiment, the influence of sampling position was evaluated by cutting five specimens from different locations along the window profile and analyzing each specimen using the same five-step depth-profiling protocol.

For the depth-profiling protocol, the crater depth after each ablation step was measured using a MarSurf CM explorer confocal microscope (Mahr, Germany). Depth profiles from three independent PVC samples were acquired. The crater depths for each ablation step were averaged, and the corresponding standard deviations were calculated.

### 2.4. Artificial Weathering Protocol

A section of industrial PVC window profile was subjected to artificial photochemical aging in a Q-SUN Xe-3 xenon arc weathering tester (Q-Lab, USA). The Q-SUN Xe-3 is a flat-array xenon arc chamber designed to reproduce the damage caused by full-spectrum sunlight and water. Aging was performed for 3000 h, corresponding to a total radiant exposure of 5.94 GJ m^−2^, under the conditions specified in Table 4. The test was run in a cyclic mode consisting of 114 min of xenon irradiation followed by a 6 min water-spray interval. Specimens were mounted at a 10° inclination from the horizontal on the sample rack.

### 2.5. Data Acquisition and Processing

For all sample types and aging states, LA-ICP-MS measurements were performed using the depth-profiling protocol described in Section “Fluence Optimization and Validation of Depth-Profiling”. For each measurement, a gas blank was recorded to correct for instrumental background. Signals were acquired for 10 s before and 30 s after the ablation, and the mean background intensities for each element were subtracted from the signals recorded during the ablation period.

Replicate measurements were performed to assess variability between specimens and measurement locations. For the comparison of laboratory samples and industrial profiles, two independent measurements were performed for each sample type: on two separately pressed sheets, two different sections of an extruded tape, and two different sections of a 6 m long industrial profile. For the comparison of different industrial profiles and calibration-vacuum conditions, measurements were performed on two different sections of the corresponding 6 m long profile. The artificially weathered specimen had dimensions of 6 × 4 cm; two sections cut from this specimen were analyzed after weathering.

For data evaluation, the median signal intensity was calculated for each element and each ablation step. The median was chosen instead of the mean because it is less sensitive to outliers arising from minor local inhomogeneities. Signal fluctuations are well known in LA-ICP-MS measurements of polymers, where heterogeneity in additive distribution and sample morphology can lead to pronounced variations in transient signals and elevated RSDs [30,31]. Depth profiles of the elemental distribution were obtained by plotting these median intensities against the corresponding ablation depth (as determined from crater measurements described in Section 2.3).

In contrast to several LA-ICP-MS studies on polymers, we did not normalize the inorganic signals to ^13^C. Previous work on polymer ablation has shown that carbon from PVC is predominantly present in the gaseous phase and in very fine particles, whereas additive elements are mainly transported as larger particulates [32]. This two-phase transport behavior and the different vaporization and ionization mechanisms of carbon compared to metals [33] imply that ^13^C is not a reliable internal standard for PVC.

For the graphical representation of depth profiles in window profiles, error bars were derived from the precision study described in Section 3.2. Consequently, the reported uncertainties include instrumental precision, as well as variability associated with local sample homogeneity and measurement location within the industrial profiles. The maximum RSD determined for each element was used to estimate the uncertainty of the median intensities: 4% for Mg and Al, 3% for Ca, and 5% for Ti across all ablation steps; for Fe, RSDs of 9.5% for the first ablation and 4.5% for ablations 2–5 were applied; for Zn, RSDs of 9.5% for the first ablation and 6% for the deeper layers were used. These RSD values were converted to standard deviations of the median intensities and plotted as error bars in the corresponding depth-profile figures.

## 3. Results

### 3.1. Fluence Optimization

To determine the optimal fluence, median signal intensity was calculated for each element along each ablation line. These median intensities were then averaged across the three replicate measurements for a given fluence, and the corresponding RSD was determined. The fluence optimization was conducted directly on industrial PVC window profiles to ensure that the chosen conditions are representative of the matrix investigated in this study. Because the absolute intensities differed strongly between elements (by up to a factor of about thirty, e.g., Al vs. Ti, reflecting their different concentrations in the PVC profile), the mean intensities and RSDs were normalized for each element. The normalized values (norm.) were then averaged (avg.) over all elements for each fluence setting and evaluated graphically to identify the fluence that provided high overall intensity and a low overall RSD.

**Figure 1:**
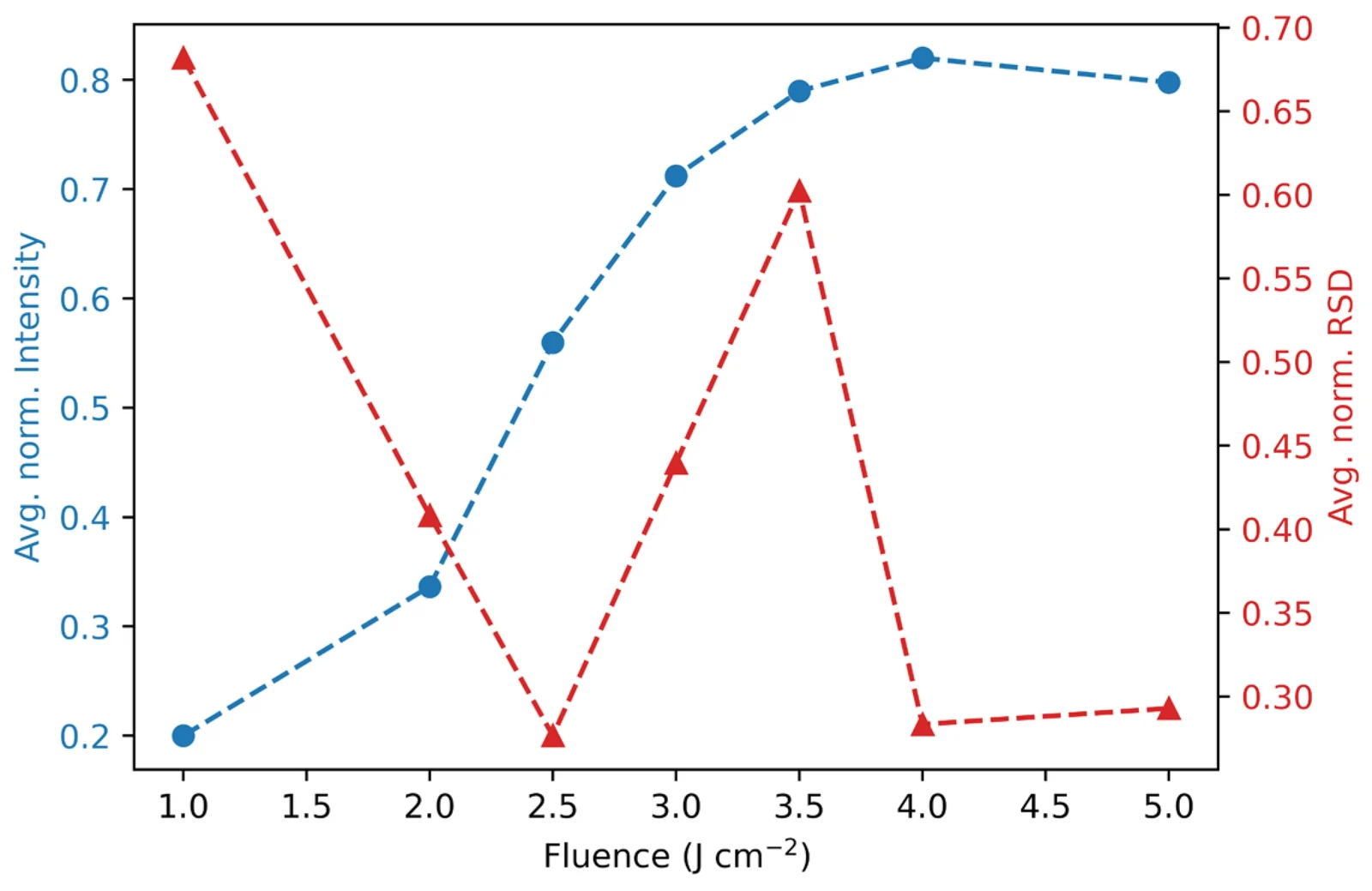
Averaged, normalized intensity (blue) and averaged, normalized RSD (red) for all measured elements (Mg, Al, Ca, Ti, and Zn) as functions of laser fluence between 1 and 5 J cm^−2^.

The resulting averaged normalized intensities (blue) and averaged normalized RSDs (red) as a function of fluence are shown in Figure 1. The intensity increases with fluence up to about 3.0 J cm^−2^ and then levels off, which suggests that at higher fluence, larger particles are generated that are less efficiently transported in the gas flow. The averaged RSD shows two local minima at 2.5 and 4.0 J cm^−2^. Starting from fluences of 3.5 J cm^−2^, a brownish discoloration of the PVC surface was observed in the vicinity of the ablation crater, indicating increased thermal impact of the laser on the matrix. As the fluence also showed an intensity plateau above 3.0 J cm^−2^, a value of 2.5 J cm^−2^ was selected for all subsequent experiments as a compromise among high signal intensity, low RSD, and preservation of the sample surface.

### 3.2. System Precision and Positional Variability

The precision of the LA-ICP-MS depth-profiling method was evaluated by repeated measurements on industrial PVC window profiles. System precision was assessed by acquiring five depth profiles at closely spaced positions on the profile, each profile consisting of five consecutive ablations. The resulting RSDs for the median intensities of Mg, Al, Ca, Ti, Fe, and Zn are summarized in Table 5. For all elements, the system-related RSD was below 3.5% for every ablation step, with typical values between 1–3%, indicating high instrumental stability of the setup.

Positional variability was assessed by analyzing five specimens cut from different locations along the same PVC window profile using the same five-step depth-profiling protocol. The corresponding RSDs are summarized in Table 6. For most elements and ablation steps, the positional RSD remained below 5%, indicating modest lateral inhomogeneity along the profile. Notable exceptions are Fe and Zn in the first ablation layer, where RSDs of up to ~9–10% were observed. Overall, the RSDs observed between different specimens from a single profile are comparatively small for most elements and depth steps, demonstrating that the sampling approach is robust.

### 3.3. Laser Crater Depth Evaluation

**Figure 2:**
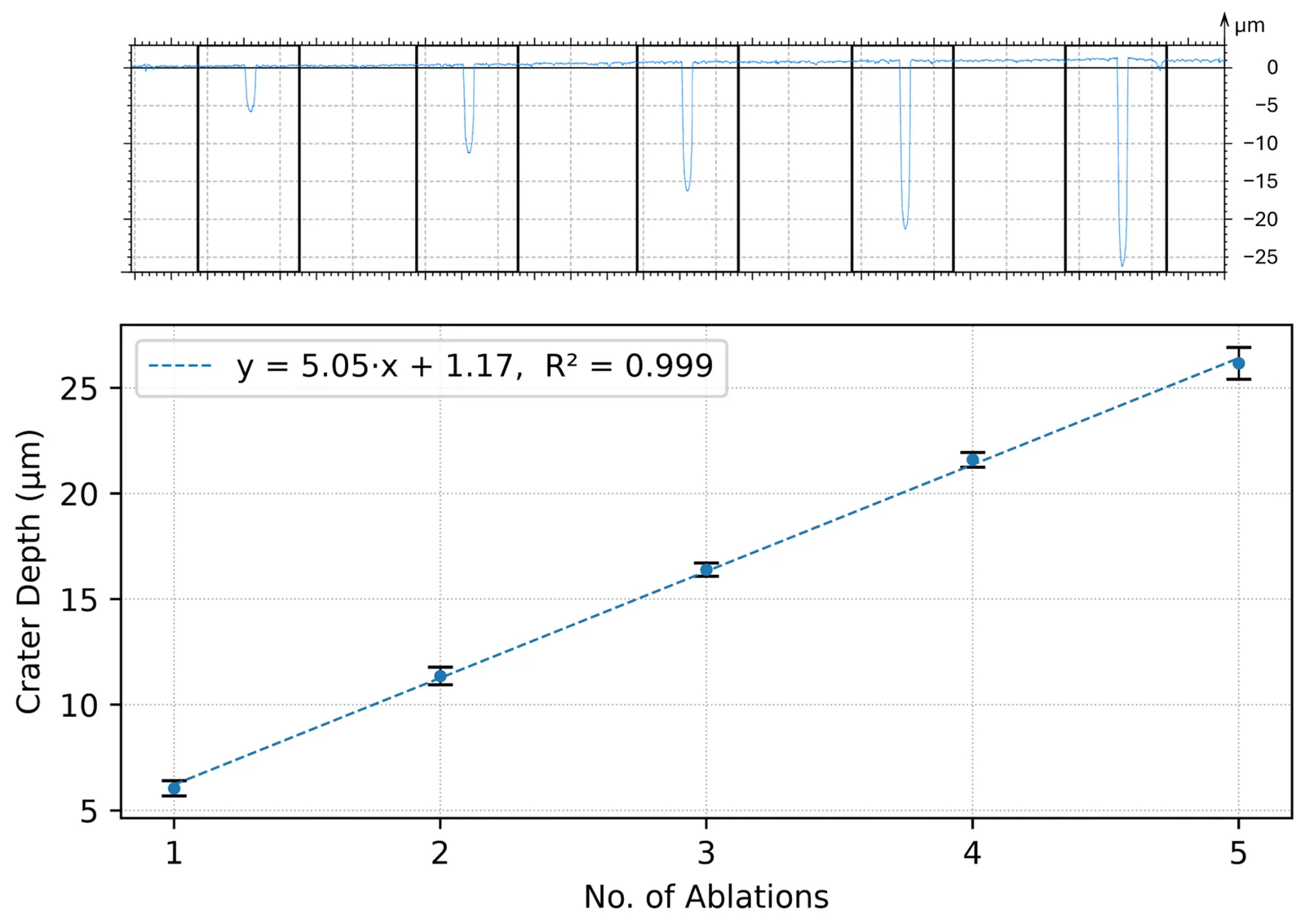
Confocal profiles (**top**) and mean laser crater depth (±2σ) (**bottom**) versus number of ablations for PVC, with linear regression.

The effectiveness of the five-step depth-profiling protocol was assessed by measuring crater depths after each successive ablation. Craters generated on three independent PVC samples were analyzed using confocal microscopy, and the depths for each ablation step were averaged; the error bars in Figure 2 represent twice the standard deviation (2σ).

The mean depth after the first ablation was 6.0 ± 0.36 µm, indicating that the initial ablation removes slightly more material than subsequent steps. From the second ablation onwards, the crater depth increased linearly with the number of ablations, with an average increment of 5.05 µm per ablation as obtained from linear regression. After five ablations, the total crater depth was 26.2 ± 0.76 µm. The lateral crater dimension remained constant at ~29.5 µm over all five ablation steps, and the crater rims appeared well defined under confocal microscopy, indicating stable focusing and beam geometry under the chosen conditions.

The standard deviations per ablation step were small (2σ ≤ 0.4 µm for ablations 1–4), demonstrating that the depth increment is highly reproducible between different samples. Although the standard deviation for the fifth ablation was somewhat higher (2σ = 0.76 µm), the overall variability in total depth remains low relative to the mean crater depth.

For the artificially aged sample, the ablation protocol was extended to six consecutive ablations to accommodate the depth range required for the weathering experiment. The crater depth after each ablation was evaluated by confocal microscopy (n = 3) using the same procedure. The measured depths were comparable to those obtained for the unaged samples, and the depth of the sixth ablation was 31.26 ± 0.64 µm. The corresponding measured depths are summarized in Table 7.

For all following evaluations of elemental distributions, signal intensities were therefore plotted as functions of ablation depth, using the mean crater depths derived from these measurements.

### 3.4. Elemental Distributions in Different Sample Types

#### 3.4.1. Comparison of Laboratory Samples and Industrial Profiles

**Figure 3:**
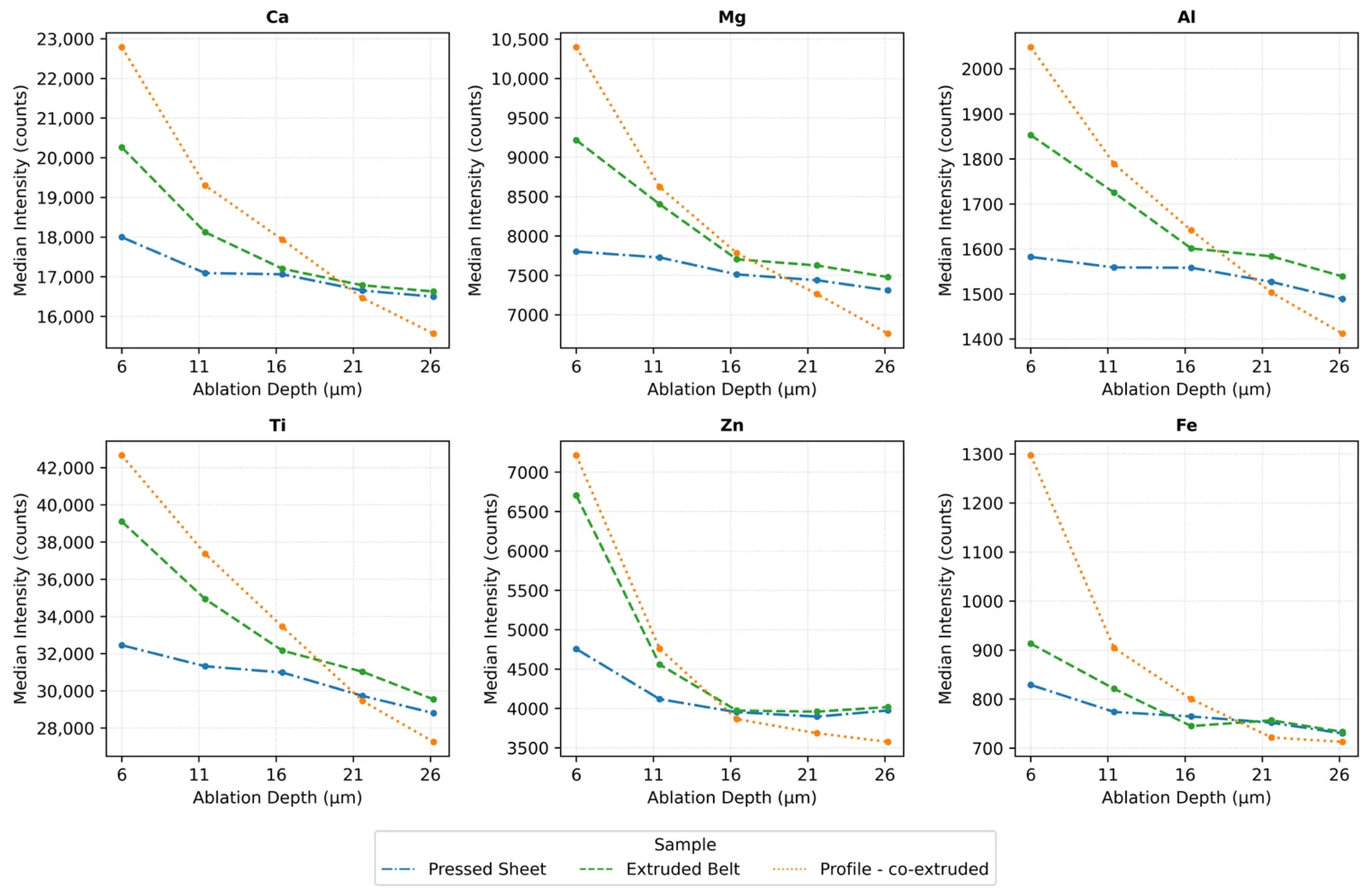
Elemental depth profiles for Ca, Mg, Al, Ti, Zn, and Fe in pressed sheet, extruded belt, and co-extruded profile.

As outlined in the Materials and Methods section, pressed sheets, extruded belts, and extruded window profiles represent typical specimen types for aging studies. Two independent measurements were performed for each sample type. These comprised two separately pressed sheets, two different sections of an extruded tape, and two different sections of an industrial profile. LA-ICP-MS depth profiling was therefore applied to these three sample types to compare their elemental distributions with depth and evaluate their suitability for aging investigations. Depth profiling revealed pronounced differences in elemental distributions among the three sample types (Figure 3). Overall, the pressed sheet showed the most homogeneous distributions with depth, whereas the co-extruded profile exhibited the strongest gradients. The replicate measurements yielded comparable elemental depth profiles for each sample type and confirmed the differences among the pressed sheet, extruded tape, and industrial window profile.

For Ca, the pressed sheet displayed a slightly increased intensity in the outer 6 µm (≈5% higher than at 26 µm), while deeper layers showed comparable intensities. In the extruded belt, Ca intensities were elevated in the near-surface region up to about 11 µm and became essentially constant from 16 µm onwards. In contrast, the co-extruded profile showed a continuous decrease in Ca intensity over the analyzed depth, with the first ablation layer exhibiting 45% higher intensity than the deepest layer.

Mg and Al were effectively homogeneous across the pressed sheet, whereas Ti showed a moderate gradient, with the surface intensity about 10% higher than the deepest layer. In the extruded belt, Mg and Al profiles resembled that of Ca. The outer 11 µm showed increased intensities (≈20% higher in 0–6 µm and ≈10% higher in 6–11 µm), followed by nearly constant values from 16 µm downward. Ti exhibited a more pronounced gradient, with the surface intensity 30% higher than for the 21–26 µm layer. From 16 µm onwards, the differences between layers decreased. In the co-extruded profile, Mg, Al, and Ti all decreased with depth, analogous to Ca.

Zn displayed the strongest depth-dependent variations. In the pressed sheet, the surface layer had an intensity 20% higher than the deeper layers, which were relatively constant. In the extruded belt, Zn intensity in the outer 6 µm was 65% higher than in the deepest layer, and still 10% higher in the 6–11 µm layer. From 16 µm downward, intensities were essentially constant. The co-extruded profile showed the largest gradient, with the surface layer exhibiting approximately twice the intensity of the deepest layer. The two outer layers in particular had elevated Zn intensities, while the inner layers decreased more gradually.

Fe in the pressed sheet and extruded belt shows behavior similar to Ca, Mg, and Al. In the pressed sheet, the surface intensity is 15% higher than in the deepest layer, while the middle layers display essentially constant intensities. In the extruded belt, Fe intensities in the outer two layers are elevated relative to the deepest layer (≈25% for the first ablation and ≈12% for the second), followed by nearly constant values from 16 µm inward. In the co-extruded profile, the Fe gradient extends to the third layer (11–16 µm), whereas the deepest two layers exhibit comparable intensities.

**Figure 4:**
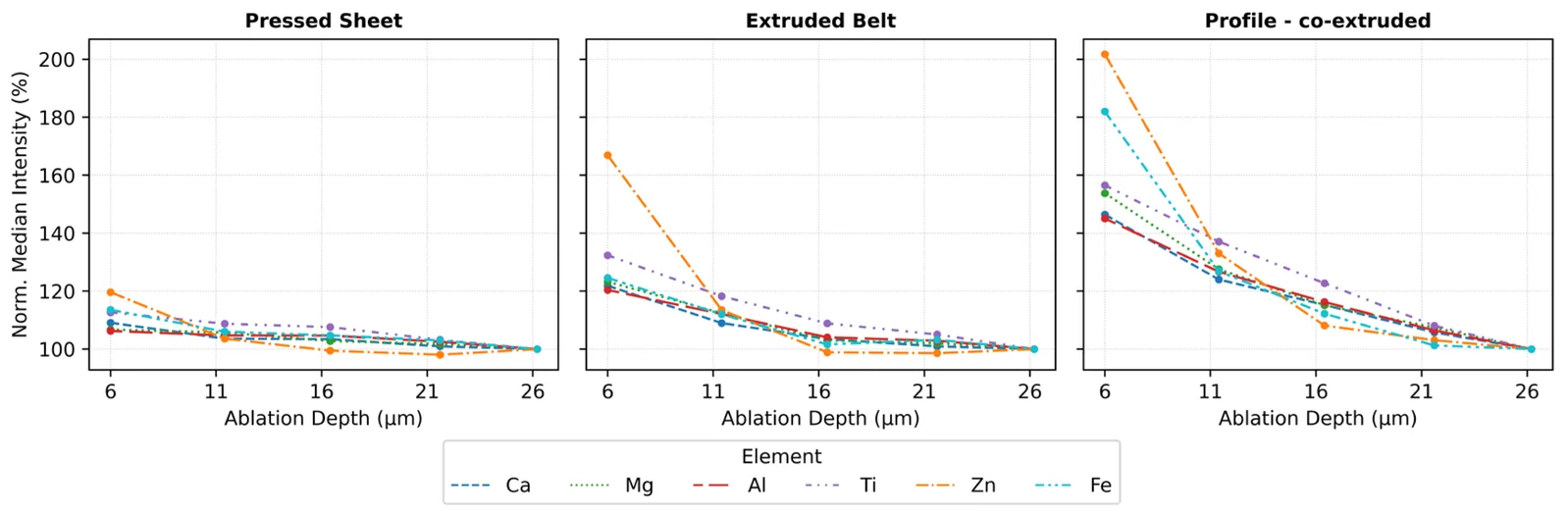
Normalized elemental depth profiles (Ca, Mg, Al, Ti, Zn, and Fe) for the three PVC sample types: pressed sheet, extruded belt, and co-extruded window profile.

To compare relative depth dependence, intensities were normalized to the deepest layer (Figure 4). In the pressed sheet, normalized profiles of Ca, Mg, Al, Ti, and Fe were very similar, while Zn showed slightly higher normalized surface intensity. In the extruded belt, normalized Ca, Mg, Al, and Fe profiles closely coincide, whereas Ti and Zn exhibited stronger near-surface enrichment. In the co-extruded profile, normalized Ca and Al profiles were nearly identical, Mg and Ti showed moderate enrichment, and Fe and Zn displayed the largest deviations from the bulk. These observations indicate that both overall density differences and element-specific surface enrichment contribute to the observed profile shapes.

#### 3.4.2. Comparison of Different Industrial Window Profiles

**Figure 5:**
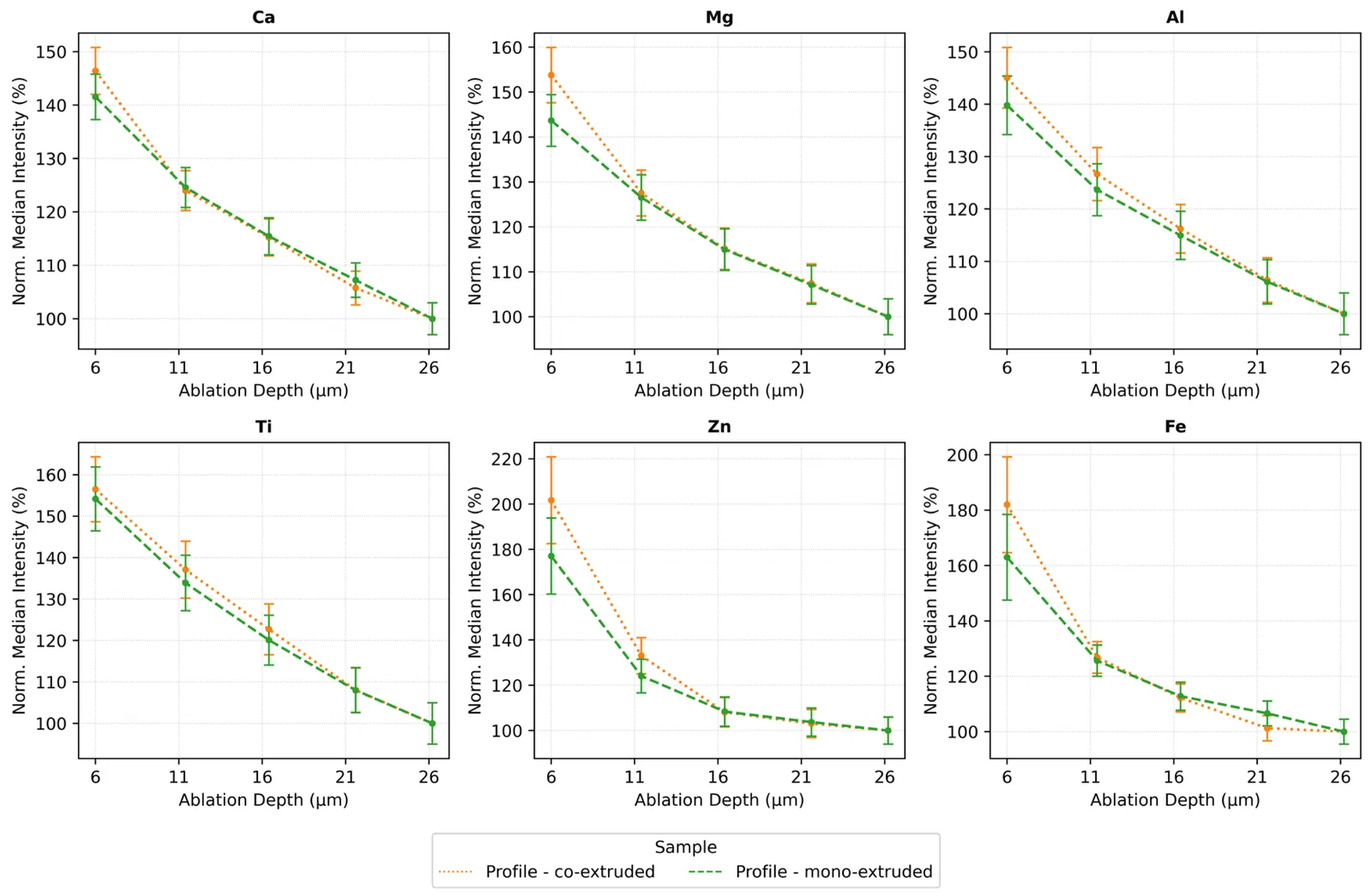
Normalized elemental depth profiles for Ca, Mg, Al, Ti, Zn, and Fe in co- and mono-extruded profiles; error bars represent the standard deviations, calculated as described in Section 2.5.

To assess whether extruder type (conical vs. parallel), extrusion mode (mono- vs. co-extrusion), and die design influence the elemental depth profiles, two industrial window profiles were examined. Measurements were performed on two different sections of the corresponding profile. For each profile, the median intensities of Ca, Mg, Al, Ti, Zn, and Fe were normalized to the intensity of the fifth ablation (26.2 µm) (Figure 5).

The normalized depth profiles of Ca, Al, and Ti for the two profiles differ only slightly, mainly in the surface layer; deeper layers are essentially identical. For Mg, the normalized surface intensity of the co-extruded profile is 10% higher than that of the mono-extruded profiles, while the deeper layers coincide. Both profiles exhibit Zn enrichment at the surface, with the effect more pronounced for the co-extruded profile (normalized surface intensity 200% vs. 180% for the mono-extruded profile). The second layer of the co-extruded profile also shows slightly higher Zn intensity (10%). For Fe, the normalized surface intensity is 180% in the co-extruded profile compared to 160% in the mono-extruded profile; with increasing depth, the profiles converge.

When the uncertainties derived from the precision study (Section 3.2) are taken into account, all measured intensities within a given depth step lie within the corresponding error bars, indicating that the observed differences between the two profiles are small compared to the measured uncertainty. Overall, the elemental depth profiles of the two specimens are therefore very similar.

#### 3.4.3. Influence of Calibration Vacuum on Elemental Depth Profiles

**Figure 6:**
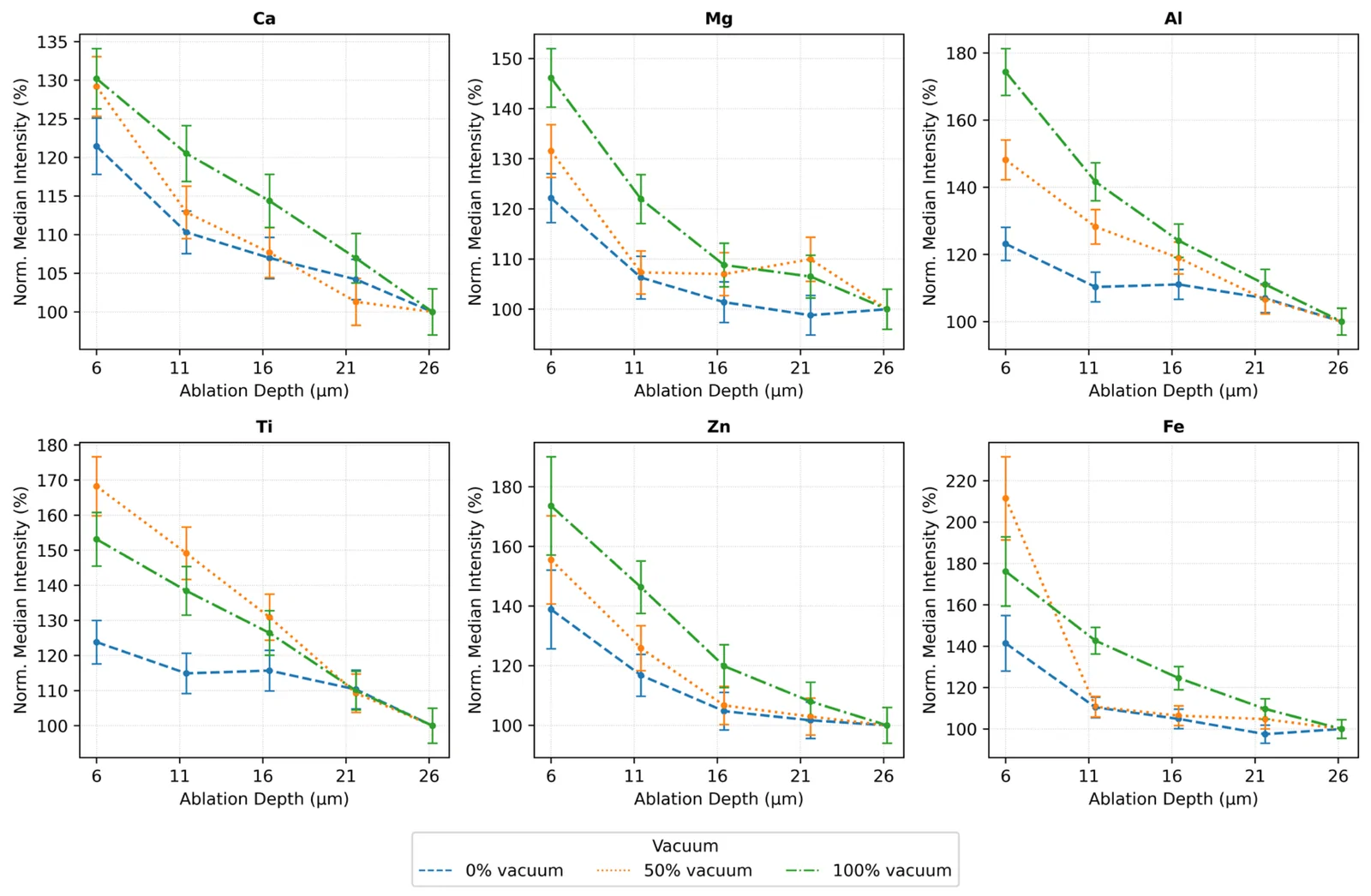
Normalized elemental depth profiles for Ca, Mg, Al, Ti, Zn, and Fe in PVC window profiles produced with different calibration vacuums (0%, 50%, and 100%); error bars represent the standard deviations, calculated as described in Section 2.5.

To investigate the influence of post-extrusion calibration on elemental depth profiles, window profiles produced with three different calibration vacuums (0%, 50%, and 100%) were analyzed. For each profile, the median intensities of Ca, Mg, Al, Ti, Zn, and Fe were normalized to the intensity at 26.2 µm (Figure 6). The three calibration-vacuum conditions were investigated using separate profiles, with two different sections measured for each condition.

For Ca, the profile calibrated with 100% vacuum exhibits the steepest gradient. The surface enrichment of Ca in the 50% and 100% vacuum profiles is comparable within the uncertainty, but the normalized intensity in the second ablation layer (6–11 µm) is higher for the 100% vacuum profile than for the other two profiles. In the case of Mg, the 100% vacuum profile shows higher normalized intensities in the outer two layers compared with the 50% and 0% vacuum profiles, while the deeper layers converge.

For Al, the gradient steepens progressively with increasing calibration vacuum. The 100% vacuum profile exhibits the highest intensities in the first and second ablation layers (up to 11 µm), followed by the 50% vacuum, whereas the 0% vacuum yields the lowest near-surface Al intensities. For Ti, normalized intensities at the surface and in the second layer for the 50% and 100% vacuum profiles lie within the measurement uncertainty and are both higher than those of the 0% vacuum.

For Zn, the most pronounced difference in surface intensity is observed between the 0% and 100% vacuum profiles, with the 100% vacuum showing the highest Zn enrichment at 0–6 µm. The uncertainty of the 50% vacuum profile overlaps with the intensities of both the 0% and 100% specimens in the surface layer, but in the second ablation layer (6–11 µm) the normalized Zn intensity is higher for the 100% vacuum profile than for 50% and 0% vacuum. For Fe, the 0% vacuum shows distinctly lower surface intensity than the 50% and 100% vacuum profiles; in the second and third ablation layers, the 100% vacuum exhibits higher normalized Fe intensities than the other two specimens.

### 3.5. Artificial Weathering of Window Profiles

Artificial weathering was performed on one window-profile specimen with dimensions of 6 × 4 cm. Two sections cut from this weathered specimen were analyzed independently by LA-ICP-MS.

**Figure 7:**
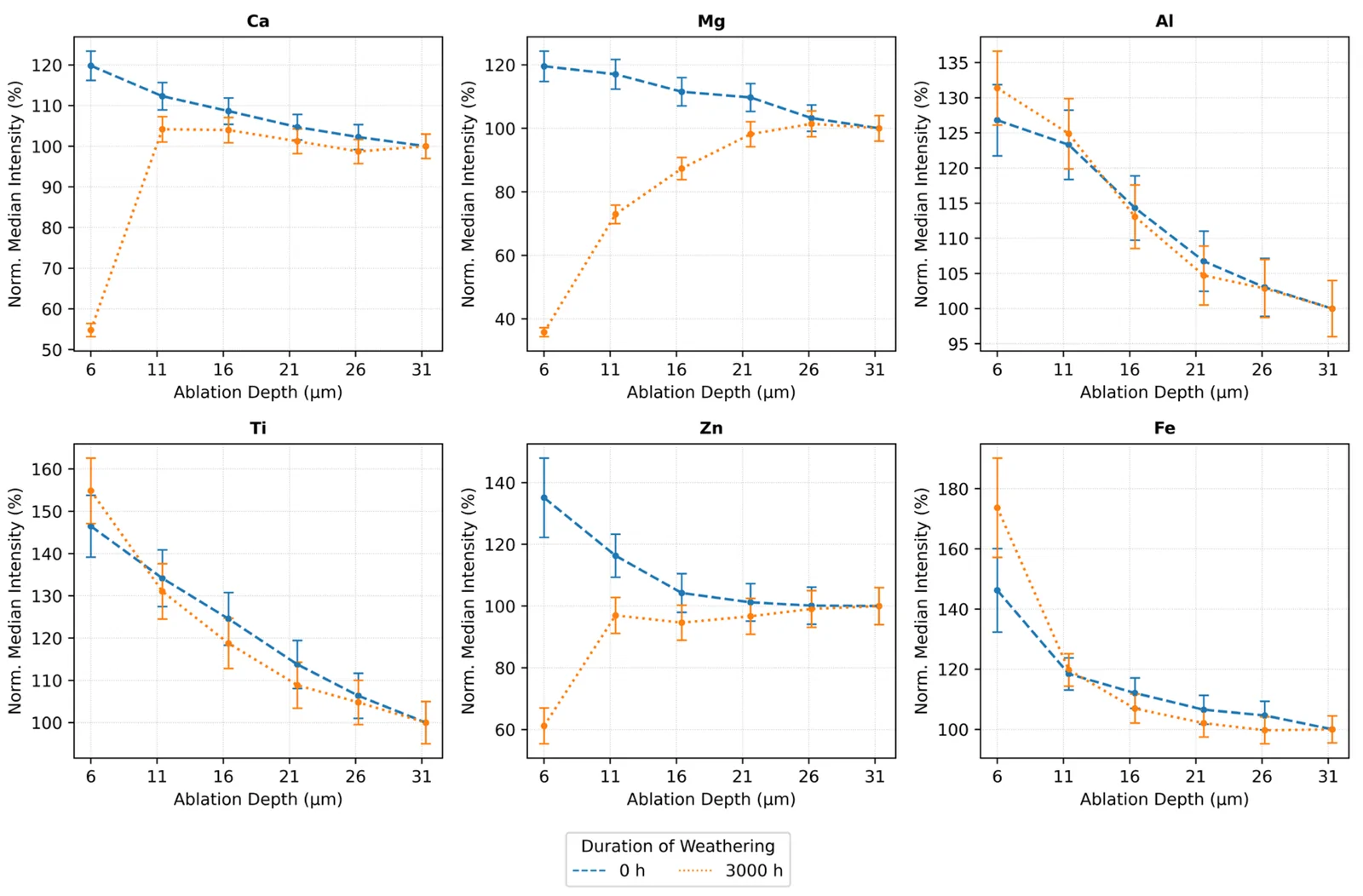
Normalized elemental depth profiles for Ca, Mg, Al, Ti, Zn, and Fe in an unaged window profile and after 3000 h of photochemical aging in the Q-SUN; error bars represent the standard deviations, calculated as described in Section 2.5.

Prior to the depth-resolved elemental analysis, the colorimetric response of the window profile was evaluated after 3000 h of artificial weathering. The results indicated minimal color degradation; specifically, the sample exhibited a slight increase in lightness with negligible shifts in chromaticity. Given the limited magnitude of these changes, the detailed colorimetric data are provided in Table S6 of the Supplementary Materials. For the subsequent evaluation of additive distribution, the two analyzed sections showed comparable depth profiles, and the absolute intensities of the measured elements were observed to converge from the sixth ablation layer onward. Therefore, all depth profiles were normalized to the intensity of the sixth ablation layer (depth of 31 µm) (Figure 7).

For Ca, a pronounced depletion was observed in the near-surface region after Q-SUN aging. In the unaged profile, the normalized intensity in the outer 6 µm was ~120%, whereas after 3000 h of weathering it decreased to ~55%. In the second layer, the normalized Ca intensity decreased only slightly (from 112% to 104%).

Mg was depleted over a larger depth range. After 3000 h of weathering, reduced Mg intensities were observed down to 21 µm. The normalized intensity in the surface layer decreased to 35%, and in the second layer to 73%. In contrast, Al showed no reduction in intensity; a slight increase at the surface remained within the measurement uncertainty. Ti likewise exhibited only minor changes within the uncertainty.

Zn showed the greatest changes in the near-surface region. In the outer 6 µm, the normalized Zn intensity decreases from 135% in the unaged state to 60% after weathering. The second layer also showed a reduction (from 116% to 97%), whereas from the third ablation onwards, the differences between unaged and aged profiles were within the measurement uncertainty.

For Fe, an apparent increase in surface intensity after weathering was observed, but this change lies within the comparatively high uncertainty of the surface ablation layer and is not considered systematic.

## 4. Discussion

### 4.1. Performance and Limitations of the LA-ICP-MS Depth-Profiling Method

The first objective of this study was to establish a matrix-adapted LA-ICP-MS protocol for depth-resolved analysis of inorganic additives and impurities in rigid PVC window profiles. The fluence optimization, precision study, and crater-depth evaluation together show that the method provides reproducible and interpretable depth profiles for the relevant additive elements.

The fluence study on industrial profiles demonstrated that signal intensity increases with fluence up to ~3 J cm^−2^ before approaching a plateau, while the averaged normalized RSD exhibits minima around 2.5 and 4 J cm^−2^. The onset of visible thermal damage (brownish discoloration) at ≥ 3.5 J cm^−2^ indicates that higher fluences compromise matrix integrity without providing further gains in intensity. Selecting 2.5 J cm^−2^ as the operational fluence therefore represents a practical compromise among sensitivity, precision, and protection of the PVC surface from thermal decomposition.

The precision and positional variability studies indicate that the main source of variability in depth profiles is the homogeneity of the sample, not instrumental instability. System-related RSDs of ~1–3% for all elements and ablation steps reflect high instrumental robustness over the timescale of a measurement sequence. Positional RSDs along a profile remain below ~5% for most elements and depths, and only Zn and Fe in the outermost layer approach 9–10%. Comparable or higher within-sample variations (11–15%) have been reported in LA-ICP-MS analysis of PVC tapes, wires, and tubing [34], which suggests that the industrial window profiles studied here are relatively homogeneous and well suited for comparative work. The elevated positional variability of Zn and Fe at the surface reflects true lateral inhomogeneity arising from migration and contamination processes. To account for this, RSDs were used as error bars in all depth-profile plots.

Crater-depth measurements confirm that the depth-profiling protocol yields a well-defined, linear depth axis. After a slightly deeper first ablation (~6 µm) associated with initial removal of surface materials and roughness, subsequent ablations remove ~5 µm of material per step, with constant lateral crater dimensions and small standard deviations. This behavior is consistent with the use of a flat-top beam profile (intensity profile) and with previous LA-ICP-MS studies on polymers that report a linear correlation between ablation depth and number of ablated layers [35]. The resulting depth range (0–31 µm) encompasses the near-surface region where photochemical damage, stabilizer depletion, and contamination effects are expected to be most pronounced.

A key methodological choice was to evaluate profiles in relative terms without internal normalization (e.g., to ^13^C). For PVC, previous work has shown that carbon is predominantly transported as gas and very fine particles, while inorganic additives are carried in larger particulates, with different vaporization and ionization mechanisms [32,33]. Using ^13^C as an internal standard in such a two-phase system can introduce matrix-dependent bias rather than correct it. In the present study, high reproducibility, carefully controlled measurement sequences, and explicit propagation of RSDs into error bars allow robust relative comparison of profile shapes among sample types, processing conditions, and aging states without carbon normalization. The main limitation is that the method yields semi-quantitative profiles rather than absolute concentrations. Further work could address this by incorporating PVC matrix-matched calibration standards.

### 4.2. Processing-Induced Differences Between Sample Types

Laboratory specimens such as pressed sheets and extruded belts are commonly used in PVC aging studies [4,12] because they can be produced quickly and economically from limited amounts of material. Industrial window profiles, by contrast, are the actual end products whose long-term performance is of interest but are seldom included in routine aging programs due to higher production effort and cost. For the PVC formulation and specimen types investigated here, the depth-resolved data show differences in both the overall signal level and the element-specific gradients, indicating differences in density, internal structure, and additive distribution arising from their respective processing routes. This raises the question of how far aging results obtained from the laboratory specimens examined within this study can be transferred to the industrial profiles under investigation, and provides a basis for assessing the validity of such extrapolations.

The pressed sheet, produced by milling and pressing, shows relatively homogeneous depth profiles for Ca, Mg, Al, and Fe, with only modest near-surface variations. Ti exhibits a modest gradient over the entire measured depth. Zn shows the most pronounced enrichment in the surface layer, while the deeper layers display essentially constant intensities. In contrast, the extruded belt and co-extruded profile display higher apparent intensities and more pronounced gradients for several elements. In the co-extruded profile, Ca, Mg, Al, and Ti all decrease continuously over the analyzed depth; Zn and Fe show particularly strong enrichments in the outer layers, with surface intensities up to about twice those in the deepest layer.

Normalizing intensities to the deepest layer within each specimen clarifies these differences. In the pressed sheet, normalized Ca, Mg, Al, Ti, and Fe profiles closely overlap, while Zn shows only a slightly elevated normalized surface intensity. In the extruded belt, normalized profiles of Ca, Mg, Al, and Fe are very similar, whereas Ti and especially Zn exhibit stronger near-surface enrichment. In the co-extruded profile, normalized Ca and Al profiles are nearly identical, Mg and Ti show moderate enrichment, and Fe and Zn display the largest deviations from the bulk. These patterns indicate that part of the difference between specimen types is a density effect, and part is element-specific surface enrichment. If the ablated volume is kept constant by the LA conditions, a higher bulk density results in a greater ablated mass and thus higher intensities for all elements in a similar way. In contrast, migration or contamination effects are element-specific and manifest as differences in the relative depth profiles of particular elements, typically as additional near-surface enrichment superimposed on the density-related signal increase.

The higher apparent signal levels observed for the extruded belt and profiles compared to the pressed sheet are consistent with differences in density and compactness arising from the respective processing conditions. Extrusion generally involves higher melt pressure, temperature, and shear than milling and pressing. For the industrial window profiles examined, additional measures such as degassing during profile extrusion and subsequent vacuum calibration—with intensive contact between the hot profile and the metal calibration tools and controlled cooling of the skin—further compact the surface layer and reduce porosity. At the same time, the hollow-chamber geometry of window profiles allows the core region to cool more slowly and relax to a greater extent, so that a density gradient with a particularly dense skin and a less compact core can be assumed. These factors provide a plausible explanation for a denser, more compact surface region in the extruded profiles studied relative to the laboratory-produced pressed sheet. From a weathering perspective, such density differences will influence the penetration depth and kinetics of moisture ingress and leaching processes in the near-surface region.

Element-specific behavior can be related to migration and contamination mechanisms. Zinc is present as zinc stearate in Ca/Zn stabilizer systems and serves as both a stabilizer and an external lubricant. External lubricants are generally characterized by lower solubility and compatibility in the polymer matrix and, in the case of zinc stearate, by a relatively low melting temperature (120–130 °C) [4,36]. Under extrusion conditions, these properties increase the mobility of zinc stearate in the melt and promote its tendency to migrate toward the surface. The pronounced Zn enrichment in the outer layers of the extruded belt and profiles, relative to the pressed sheet, is consistent with this external-lubricant behavior of zinc stearate.

Ti, present as TiO_2_ pigment, shows moderate near-surface enrichment in the extruded belt and profiles relative to the pressed sheet. Although TiO_2_ itself is not highly mobile, its distribution is affected by dispersion, melt rheology, and flow behavior in dies and calibration units. Migrating lubricants and other mobile species can transport pigment particles toward interfaces, contributing to TiO_2_-rich skin layers and, in more extreme cases, to deposits on dies and calibration tools (“plate-out”) described in industrial practice [15,37]. Such pigment enrichment at the surface enhances UV absorption and scattering in the near-surface region, thereby shielding the underlying PVC and masking discoloration more effectively than in specimens with more homogeneous pigment distributions [25,26,27]. But at the same time, TiO_2_ is photoactive.

Fe is not deliberately added but is introduced as an impurity in raw materials (e.g., chalk [38], stabilizer) and during processing via contact with metal surfaces [16]. Its elevated normalized intensity in the outer two layers of the extruded belt and profiles and nearly constant deeper values point to surface contamination rather than bulk compositional differences. In PVC, the presence of transition metals at the surface is particularly relevant because they can catalyze oxidative degradation through hydroperoxide decomposition during photochemical aging [6,16].

Taken together, the Zn, Ti, and Fe profiles demonstrate that, for the formulation and processing conditions investigated in this study, the chemical environment in the first tens of micrometers of the industrial window profiles differs markedly from that in the laboratory specimens. The investigated profiles exhibit a Zn-rich, TiO_2_-rich, and metal-contaminated skin layer superimposed on a denser surface structure, whereas the pressed sheet is more homogeneous and less contaminated. Similar surface-biased enrichments of metallic species after extrusion or molding have been reported in LA-ICP-MS studies on other industrial polymers [31,39], indicating that such processing-related segregation is a general phenomenon, rather than specific to the materials examined here. Because photochemical damage, stabilizer depletion, pigment-mediated UV shielding, and metal-catalyzed oxidation are all concentrated in this near-surface region, aging results obtained from the laboratory specimens examined in this study should not be transferred directly to the window profiles under investigation without considering their different initial additive and impurity distributions. The depth-resolved LA-ICP-MS data presented here provide a basis for quantifying these processing-induced differences and for evaluating how they affect the interpretation and design of PVC aging studies.

### 4.3. Influence of Extrusion Mode and Calibration Vacuum

The comparison of the mono-extruded and co-extruded window profiles shows that, for the formulation and processing conditions examined, differences in extruder type (conical vs. parallel) and extrusion mode (mono vs. co-extrusion) have only minor effects on the overall shapes of elemental depth profiles. Normalized Ca, Al, and Ti profiles are nearly identical between the two profiles, and Mg, Zn, and Fe differ only modestly in the outer layer, with all differences lying within or close to the measurement uncertainty. This indicates that, within the processing window and profile types examined here, extruder type and co-extrusion do not fundamentally alter the bulk distribution of stabilizer, fillers, and pigments. Aging trends observed on one of these profile types may therefore be comparable with those of the other profile types, provided that each aged specimen is compared with an appropriate unaged reference.

In contrast, the calibration vacuum shows a clear and systematic influence on near-surface elemental distributions. Increasing the vacuum from 0% to 50% and 100% produces progressively steeper gradients and higher normalized near-surface intensities for Ca, Mg, and Al, with similar tendencies for Zn, Ti, and Fe. Under full vacuum, the skin layer exhibits the most pronounced enrichment of these elements, extending into the second and, in some cases, the third ablation layers. These observations support the working hypothesis that the surface region of window profiles is more compacted than the core and that this compaction is related to the conditions during vacuum calibration. Vacuum calibration is applied in industrial practice to overcome die swell and thus obtain dimensionally stable profiles. At the same time, it increases melt compaction and contact with the calibration tools at the surface. The depth-resolved elemental profiles measured here are consistent with a denser, more highly packed skin layer under full vacuum, superimposed on a less compact core.

### 4.4. Implications for Weathering Studies and Transferability of Laboratory Results

A central motivation for this work was to evaluate whether additive distributions in the laboratory specimens under investigation can be used to predict aging behavior in the industrial PVC window profiles. For the formulation and specimen types examined, the LA-ICP-MS results indicate that this is only partially the case. The pressed sheet and extruded belt differ from the investigated window profiles in density, morphology, and the spatial distribution of stabilizer, pigments, and impurities. In particular, the pronounced near-surface enrichment of Zn, Ti, and Fe in the extruded profiles—a consequence of extrusion and calibration processes—is largely absent or much weaker in the laboratory specimens.

These differences may have direct implications for photochemical aging and the interpretation of weathering studies. For the samples examined in this study, the Zn-rich skin in profiles provides locally elevated stabilizer content at the surface, which may influence the kinetics of stabilizer consumption, migration, and leaching during UV/water exposure. Fe, present as a surface-biased contaminant, introduces transition metals that can catalyze oxidative degradation via hydroperoxide decomposition under photochemical conditions, accelerating dehydrochlorination and chain scission in the near-surface region [16,40]. These mechanistic implications are plausible based on the measured elemental distributions and the known function of the relevant additives and impurities. Consequently, the laboratory specimens, with lower surface Fe contamination and weaker Zn enrichment, may not reproduce the same aging response as the investigated industrial profiles under otherwise similar aging conditions.

The near-surface enrichment of TiO_2_ in the investigated industrial profiles may also influence photochemical aging. TiO_2_-rich skin can have a dual effect on PVC degradation. On the one hand, they enhance UV absorption and scattering in the first few tens of micrometers, reducing the penetration depth of photochemical damage and masking color changes more effectively than in specimens with more homogeneous pigment distribution. On the other hand, TiO_2_ is photoactive: UV photons absorbed by the pigment generate charge separation (electrons and holes), a fraction of which react with oxygen and water to produce highly reactive OH and OOH radicals at the TiO_2_ surface. These radicals attack unsaturation in the polymer, promote the formation of oxidation products—mainly carbonyl species—and, in the presence of water, contribute to erosion and removal of low-adhesion surface layers. Surface treatments on TiO_2_ can suppress this photochemistry to some extent, but cannot eliminate it entirely [16,27,28]. Thus, compared with the laboratory specimens, a TiO_2_-rich skin layer in the investigated window profiles may simultaneously improve UV shielding and visual masking while also providing sites for photooxidative degradation at the pigment-polymer interface.

Taken together, the density gradients and additive/impurity distributions observed in the industrial window profiles mean that the near-surface region of real products is both structurally and chemically distinct from that of the investigated laboratory specimens. Because photochemical damage, stabilizer depletion, pigment-mediated UV interaction, and metal-catalyzed oxidation are all concentrated in this region, aging results obtained on the pressed sheet or extruded belt examined in this study should only be extrapolated to the window profiles with caution. The depth-resolved LA-ICP-MS data presented here provide a quantitative basis for assessing these processing-induced differences and for evaluating how they can affect the design and interpretation of PVC aging studies. In practice, this suggests that industrial profiles processed under representative conditions should be included in weathering programs, or at least that differences in initial additive and impurity distributions between laboratory specimens and profiles should be characterized and taken into account when transferring laboratory results to real products.

### 4.5. Artificial Weathering—Depth-Resolved Depletion of Elements Associated with Stabilizers, Co-Stabilizers, and Fillers

The LA-ICP-MS depth profiles before and after Q-SUN xenon arc exposure provide direct evidence of how inorganic components are consumed and leached during artificial weathering in the near-surface region of PVC window profiles. LA-ICP-MS measures elemental signals and can therefore resolve changes in the depth distribution of elements, but cannot identify the specific chemical species or phase in which those elements are present. Because the profiles were normalized to a deeper reference layer (6th ablation), the observed changes can be interpreted in terms of relative depletion and redistribution rather than absolute loss.

The pronounced decrease in Ca signal in the outer 6 µm after 3000 h Q-SUN aging—from 120% in the unaged state to 55%—indicates leaching of water-soluble compounds. That the second layer (6–11 µm) shows only a slight decrease in normalized Ca intensity (from 112% to 104%) indicates that this process is largely confined to the outermost region, where both HCl concentration and water contact are highest. Mg shows a more extended depletion zone than Ca, with normalized intensities reduced down to 21 µm after weathering (first layer down to 35%, second to 73%). This suggests that Mg-containing phases closer to the surface are actively involved in HCl neutralization and that their reaction products are leached over a larger depth range. Zn shows the greatest relative change in the outer layers, with normalized intensity in the first 6 µm decreasing from 135% to 60% after Q-SUN aging and a smaller reduction in the second layer (from 116% to 97%). Al and Ti depth profiles show no systematic depletion—and only minor, uncertainty-limited changes at the surface under Q-SUN conditions. Fe shows an apparent increase in intensity in the outermost layer after aging, but this change lies within the relatively high uncertainty associated with the first ablation and is not interpreted as a systematic effect.

The observed trends can be interpreted in the context of established degradation and stabilization mechanisms in PVC. Photochemical dehydrochlorination generates HCl, which can react with acid-scavenging or stabilizing components. Depending on the phases present, these components may include calcium carbonate, calcium stearate or other carboxylates, magnesium hydroxide, Mg- and Al-containing hydrotalcites, and Zn stearate or other Zn-containing stabilizer species. Such reactions may produce chloride-containing or otherwise more water-soluble products that can be removed during the repeated water-spray cycles. Similar near-surface depletion of Ca and Zn and formation of chloride species after UV/water exposure have been reported by other authors using X-ray photoelectron spectroscopy (XPS) [17,28], supporting this interpretation. LA-ICP-MS extends these observations by resolving the depth range over which Ca and Zn-based components are consumed and leached, rather than providing only an integral surface signal. Hydrotalcite, a layered double hydroxide containing Mg and Al, is expected to behave differently. It acts as an HCl sink by incorporating chloride into its structure rather than forming readily leachable salts [41,42]. At the same time, Al in the formulation is not exclusively present in hydrotalcite, but also in the coating of the TiO_2_ pigment, so the Al signal reflects contributions from both phases. The fact that Al depth profiles show no systematic depletion—and only minor, uncertainty-limited changes at the surface—indicates that neither hydrotalcite nor the TiO_2_ coating is leached under the Q-SUN conditions.

These mechanisms provide a chemically plausible explanation for the measured elemental changes, but the relative contribution of the individual phases cannot be established by LA-ICP-MS alone. Specific identification of the compounds and reaction products would require complementary methods, such as infrared spectroscopy or XPS. Thus, the LA-ICP-MS results provide depth-resolved evidence for weathering-induced changes in elemental composition, while the proposed chemical pathways are interpretations based on the known chemistry of PVC stabilization and degradation.

The depth-resolved depletion of Ca, Mg, and Zn in the near-surface region, together with the unchanged Al and Ti profiles, indicates that some components are consumed and partially leached under Q-SUN conditions, whereas others are less affected. In all cases, the loss of these inorganic components directly reduces the local stabilizer capacity in the region where photochemical damage is concentrated. As the initial stabilizer-rich skin layer is depleted, the surface becomes less buffered against ongoing dehydrochlorination and oxidation, and subsequent degradation processes are expected to accelerate.

At the same time, continuous leaching increases porosity and micro-roughness in the outer layers, making deeper regions more accessible to UV, oxygen, water, and leaching processes. This effect is particularly evident in the Mg profile, where depletion extends down to 21 µm, indicating that water and reactive species can penetrate beyond the immediate surface and interact with stabilizer phases at greater depths. For Ca, analogous deeper changes are less pronounced in the normalized data because of its higher overall concentration; differences must be larger before they become clearly visible. Nevertheless, the strong Ca depletion in the outermost layer and the partial change in the second layer suggest that Ca-based phases in deeper regions will also be affected as exposure progresses. These findings are consistent with more general degradation and erosion mechanisms described in the literature for PVC under UV and water exposure, where cyclic irradiation and water spraying lead to mechanical degradation and erosion concentrated around localized defects rather than a generalized removal of the entire surface layer. In such cases, the superficial layer is only partially eroded, and deeper, more uniform regions are uncovered locally rather than over the whole surface [28]. The depth-resolved LA-ICP-MS data obtained here are consistent with this picture: depletion and leaching of Mg-, Ca-, and Zn-containing compounds are clearly observed in the outer ~20 µm; porosity and accessibility of deeper layers increase, but the profiles do not indicate complete removal of the entire superficial layer.

Overall, the combination of depth-resolved inorganic profiling and existing mechanistic insights from the literature suggests that, under the Q-SUN conditions used here, PVC window profiles undergo a gradual transformation of the near-surface region: stabilizers, co-stabilizers, and fillers are consumed and leached; porosity increases; and localized erosion exposes deeper material that is initially less defective but progressively becomes involved in aging as exposure continues.

### 4.6. Integrating LA-ICP-MS with Other Techniques and Future Direction

The artificial weathering results demonstrate that LA-ICP-MS can monitor changes in the depth distribution of inorganic constituents during UV/water exposure. In the investigated PVC window profile, decreases in the Ca, Mg, and Zn signals and the relative stability of the Al and Ti signals provided depth-resolved evidence of weathering-induced changes in elemental composition. These observations are consistent with changes involving Ca-, Mg-, and Zn-containing stabilizer, co-stabilizer, and filler phases. However, LA-ICP-MS does not identify the chemical species or phases containing the measured elements and therefore cannot, by itself, distinguish between the individual contributions of compounds such as calcium carbonate, calcium-containing stabilizer, magnesium hydroxide, hydrotalcite, or zinc-containing stabilizer. The proposed chemical pathways should consequently be regarded as interpretations based on the known chemistry of PVC stabilization and degradation.

The present study also does not provide an independent assessment of degradation of the PVC polymer matrix. To relate the observed elemental changes to the overall aging state of the material, LA-ICP-MS should therefore be combined with complementary spectroscopic and surface-analytical methods. Techniques such as XPS, IR, Raman, and UV/Vis can provide complementary information on carbonyl formation, conjugated polyenes, dehydrochlorination, and surface chemistry evolution. Together, these approaches can clarify how stabilizer depletion and pigment behavior correlate with changes in color and gloss, as well as with the onset and progression of structural damage in PVC profiles.

Further work could extend the present approach in the following ways:

• Monitoring the time-dependence of depletion profiles;
• Correlating elemental depth profiles with independent indicators for polymer degradation;
• Comparing different stabilizer packages and filler or pigment grades.

Such studies would help establish depth-resolved inorganic profiling as a complementary technique for mechanistic understanding and long-term assessment of PVC window profiles and related rigid PVC products.

## 5. Conclusions

A matrix-adapted LA-ICP-MS protocol was established for rigid PVC that yields reproducible, depth-resolved elemental profiles over the first ~25 µm of window profiles. Optimization of laser fluence, validation of instrumental precision, and crater-depth measurements confirmed that the method can reliably resolve variations in the distribution of stabilizer elements, fillers, pigments, and metallic impurities.

A comparative profiling study conducted in this research on a pressed sheet, an extruded belt, and industrial window profiles demonstrated that processing generates pronounced differences in near-surface additive and impurity distributions. For the PVC formulation and processing conditions examined, the extruded profiles exhibited a denser skin layer enriched in Zn and Ti and contaminated with Fe, while calibration vacuum influenced the extent of near-surface enrichment. These processing-induced gradients are less pronounced in the laboratory specimens investigated. Accordingly, aging results obtained from the tested laboratory specimens should not be transferred directly to the investigated window profiles without accounting for differences in initial additive distributions and processing history. Within the set of industrial profiles examined, profiles produced from the same formulation exhibited similar elemental depth profiles, suggesting that comparison between such profiles is feasible when their processing history and initial additive distributions are comparable. Whether this applies more broadly to other formulations, extrusion lines, or profile designs requires additional investigation.

Artificial xenon arc UV/water exposure caused depth-resolved depletion of Ca-, Mg-, and Zn-containing components in the near-surface region, consistent with HCl neutralizing and leaching of soluble chlorides, while Al and Ti remained effectively constant. The combination of LA-ICP-MS with complementary spectroscopic methods offers a promising route to fully connect depth-resolved inorganic changes to macroscopic degradation, and to improve the mechanistic understanding and durability assessment of PVC window profiles and related rigid PVC products.

## Figures and Tables

**Table 1:** Laser ablation operating conditions.

Variable	
Laser output energy (mJ)	5
Fluence (J cm^−2^)	2.5 ^a^
Laser repetition rate (Hz)	30
Laser beam geometry	Circular
Spot size (µm)	25
Scan mode	Fixed speed
Stage scan speed (µm s^−1^)	50
Helium gas flow rate 1/2 (L min^−1^)	0.5/0.3

^a^ optimized fluence for rigid PVC.

**Table 2:** ICP-MS operating conditions.

Variable	
RF power (W)	1500
Plasma gas flow rate (L min^−1^)	15
Auxiliary gas flow rate (L min^−1^)	0.9
Nebulizer gas flow rate (L min^−1^)	1.25
Helium collision gas flow rate (mL min^−1^)	4.5
Integration time per isotope (s)	0.1
Measured isotopes	^24^Mg, ^27^Al, ^44^Ca, ^47^Ti, ^56^Fe, ^66^Zn

**Table 3:** Approximate mass fraction of elements in a PVC window formulation.

Element	Mass Fraction (mg kg^−1^)
Mg	2000
Al	700
Ca	38,000
Ti	15,000
Zn	600

**Table 4:** Q-SUN Xe-3 operating conditions.

Variable	
Radiation source	Xenon arc lamp X-1800
Filter	Daylight Q
Irradiance 290–800 nm (W m^−2^)	550
Relative Humidity (%)	65
Black standard temperature (°C)	60

**Table 5:** Instrumental precision of LA-ICP-MS measurements on PVC window profiles, expressed as RSD (%) for five consecutive ablations.

	System Precision Expressed as RSD (%) ^a^					
No. of Ablations	Mg	Al	Ca	Ti	Fe	Zn
1	3.4	2.7	3.1	2.9	2.5	1.4
2	1.2	2.2	1.9	2.4	2.0	1.1
3	1.5	1.8	2.0	1.8	0.9	0.9
4	2.5	1.5	2.1	1.5	1.7	1.1
5	2.5	2.1	1.9	1.7	2.0	2.3

^a^ n = 5.

**Table 6:** Positional variability of LA-ICP-MS measurements on PVC window profiles, expressed as RSD (%) for five consecutive ablations at different sampling positions.

	Positional Variability Expressed as RSD (%) ^a^					
No. of Ablations	Mg	Al	Ca	Ti	Fe	Zn
1	1.9	2.5	2.2	2.5	9.5	9.4
2	4.1	3.5	1.6	4.2	2.4	5.7
3	2.1	2.7	2.2	2.9	4.6	5.5
4	1.6	3.9	2.2	4.2	4.0	5.3
5	3.5	3.8	2.0	4.9	3.6	4.9

^a^ n = 5.

**Table 7:** Mean laser crater depth (±2σ) of the artificially aged sample.

No. of Ablations	Ablation Depth of the Artificially Aged Sample (µm)
1	6.46 ± 0.28
2	12.01 ± 0.30
3	17.03 ± 0.18
4	21.74 ± 0.91
5	26.46 ± 0.66
6	31.26 ± 0.64

## Data Availability

The original contributions presented in this study are included in the article/Supplementary Material. Further inquiries can be directed to the corresponding author.

## Supplementary Materials

The following supporting information can be downloaded at: https://www.mdpi.com/article/10.3390/polym18212626/s1, Table S1: Raw materials used for the dry-blend production; Table S2: Two-roller mill LRM-SC-110/T3E (Labtech Engineering, Thailand) operating conditions; Table S3: Hydraulic press LP-S20 (Labtech Engineering, Thailand) operating conditions; Table S4: Single-screw extruder operating conditions; Table S5: Process parameters of extrusion for window profiles; Table S6: Color shift in the CIELAB color space after 3000 h Q-SUN weathering.

## Abbreviations

The following abbreviations are used in this manuscript:

PVC	Poly(vinyl chloride)
LA	Laser ablation
ICP-MS	Inductively coupled plasma mass spectrometry
IR	Infrared spectroscopy
MS/MS	Tandem mass spectrometry
RSD	Relative standard deviation
SRM	Standard reference material
norm.	normalized
avg.	averaged
n	Number of repetitive measurements
2σ	Twice the standard deviation
XPS	X-ray photoelectron spectroscopy

## References

[1] Plastics Europe (2025). Plastics—The Fast Facts 2025.

[2] The European Council of Vinyl Manufacturers PVC in Building and Construction. https://pvc.org/applications/building-and-construction/.

[3] Rewindo GmbH Kunststofffensterrecycling in Zahlen 2025. https://rewindo.de/infomaterial/.

[4] Schiller M. (2022). PVC Additives.

[5] Wypych G. (2020). PVC Formulary.

[6] Girois S. (1999). Effect of iron compounds (oxide, salt, or complexes) on poly(vinyl chloride) thermal and photochemical stability. J. Vinyl Addit. Technol..

[7] Al-Dossary A.K., Gilbert M., Hitt D. (2009). Evaluating PVC Degradation Using UV and Raman Spectroscopies. AMR.

[8] Kip B.J., van Aaken S.M., Meier R.J., Williams K.P.J., Gerrard D.L. (1992). Considerations for Raman spectroscopic determination of polyene length distribution in degraded poly(vinyl chloride). Macromolecules.

[9] Novikov V.S., Kuznetsov S.M., Kuzmin V.V., Prokhorov K.A., Sagitova E.A., Darvin M.E., Lademann J., Ustynyuk L.Y., Nikolaeva G.Y. (2021). Raman Scattering Study of Natural and Synthetic Compounds Containing Polyene Chains. Dokl. Phys..

[10] Gaumet S., Gardette J.-L. (1991). Photo-oxidation of poly(vinyl chloride): Part 2—A comparative study of the carbonylated products in photo-chemical and thermal oxidations. Polym. Degrad. Stab..

[11] Amar Z., Chabira S., Sebaa M., Ahmed B. (2019). Structural Changes Undergone During Thermal Aging and/or Processing of Unstabilized, Dry-blend and Rigid PVC, Investigated by FTIR-ATR and Curve Fitting. ACSM.

[12] Anton-Prinet C., Mur G., Gay M., Audouin L., Verdu J. (1998). Photoageing of rigid PVC—I. Films containing thermal stabiliser. Polym. Degrad. Stab..

[13] Braun D., Sonderhof D. (1985). Assignment of UV-absorption maxima of degraded PVC. Polym. Bull..

[14] Frye A.H., Horst R.W. (1959). The mechanism of poly(vinyl chloride) stabilization by barium, cadmium, and zinc carboxylates. I. Infrared studies. J. Polym. Sci..

[15] Varshney N., Gilbert M., Walon M., Schiller M. (2012). Plate-out in PVC extrusion. II. Lubricant effects on the formation of die plate-out in lead-based rigid PVC formulations. J. Vinyl Addit. Technol..

[16] Pimentel Real L.E. (2023). Weathering of Polymers and Plastic Materials.

[17] Pimentel Real L.E., Ferraria A.M., Botelho do Rego A.M. (2008). Comparison of different photo-oxidation conditions of poly(vinyl chloride) for outdoor applications. Polym. Test..

[18] Villaseñor Á., Sánchez R., Boccongelli M., Todolí J.-L. (2021). Localized Quantitative Analysis of Polymeric Films through Laser Ablation-Inductively Coupled Plasma Mass Spectrometry. Polymers.

[19] Deiting D. (2017). Matrixunabhängige Elementbestimmung in Polymeren Mittels Massenspektrometrie mit Induktiv Gekoppeltem Plasma nach Laserablation. Ph.D. Dissertation.

[20] Deiting D., Börno F., Hanning S., Kreyenschmidt M., Seidl T., Otto M. (2016). Investigation on the suitability of ablated carbon as an internal standard in laser ablation ICP-MS of polymers. J. Anal. At. Spectrom..

[21] El Hadri H., Gigault J., Mounicou S., Grassl B., Reynaud S. (2020). Trace element distribution in marine microplastics using laser ablation-ICP-MS. Mar. Pollut. Bull..

[22] Brunnbauer L., Mayr M., Larisegger S., Nelhiebel M., Pagnin L., Wiesinger R., Schreiner M., Limbeck A. (2020). Combined LA-ICP-MS/LIBS: Powerful analytical tools for the investigation of polymer alteration after treatment under corrosive conditions. Sci. Rep..

[23] Wolf R.E., Thomas C., Bohlke A. (1998). Analytical determination of metals in industrial polymers by laser ablation ICP-MS. Appl. Surf. Sci..

[24] Voss M., Nunes M.A.G., Corazza G., Flores E.M.M., Müller E.I., Dressler V.L. (2017). A new approach to calibration and determination of selected trace elements in food contact polymers by LA-ICP-MS. Talanta.

[25] Anton-Prinet C., Mur G., Gay M., Audouin L., Verdu J. (1998). Photoageing of rigid PVC—IV. Effects of titanium dioxide. Polym. Degrad. Stab..

[26] Yang T.-C., Noguchi T., Isshiki M., Wu J.-H. (2014). Effect of titanium dioxide on chemical and molecular changes in PVC sidings during QUV accelerated weathering. Polym. Degrad. Stab..

[27] Gao A.X., Bolt J.D., Feng A.A. (2008). Role of titanium dioxide pigments in outdoor weathering of rigid PVC. Plast. Rubber Compos..

[28] Pimentel Real L.E., Ferraria A.M., Botelho do Rego A.M. (2007). The influence of weathering conditions on the properties of poly(vinyl chloride) for outdoor applications. An analytical study using surface analysis techniques. Polym. Test..

[29] Kerkeling S., Elfers B.-M., Kreyenschmidt M., Fittschen U.E.A. (2026). Development of a multi-element quantification method for technical polymers containing refractory oxides after microwave-assisted digestion using HBF 4. J. Anal. At. Spectrom..

[30] Stehrer T., Heitz J., Pedarnig J.D., Huber N., Aeschlimann B., Günther D., Scherndl H., Linsmeyer T., Wolfmeir H., Arenholz E. (2010). LA-ICP-MS analysis of waste polymer materials. Anal. Bioanal. Chem..

[31] Hendriks L., Egger M., Mitrano D.M. (2025). Investigating metal distribution patterns in pristine and ocean-weathered plastics using LA-ICP-TOFMS. J. Anal. At. Spectrom..

[32] Todolí J.-L., Mermet J.-M. (1998). Study of polymer ablation products obtained by ultraviolet laser ablation—Inductively coupled plasma atomic emission spectrometry. Spectrochim. Acta B Spectrosc..

[33] Frick D.A., Günther D. (2012). Fundamental studies on the ablation behaviour of carbon in LA-ICP-MS with respect to the suitability as internal standard. J. Anal. At. Spectrom..

[34] Bruin-Hoegée M.d., Corzo R., Zoon P.D., Vergeer P., Schoorl J., van der Schans M.J., Noort D., van Asten A.C. (2024). Evaluating the strength of evidence of elemental profiling of polymers with LA-ICP-MS. Forensic Chem..

[35] Willner J., Brunnbauer L., Quarles C.D., Nelhiebel M., Larisegger S., Limbeck A. (2023). Development of a simultaneous LA-ICP-MS & LIBS method for the investigation of polymer degradation. J. Anal. At. Spectrom..

[36] Wypych G. (2020). PVC Degradation and Stabilisation.

[37] Gilbert M., Varshney N., van Soom K., Schiller M. (2008). Plate-out in PVC extrusion. I. Analysis of plate-out. J. Vinyl Addit. Technol..

[38] Wypych G. (2021). Handbook of Fillers.

[39] Spanu D., Botta L., Carnati S., Grande T., Kalčíková G., Nizzetto L., Pozzi A., Šupraha L., Binda G. (2026). Investigating Plastic-Metal Interactions in Aquatic Environments Using Laser Ablation ICP-MS and Chemical Markers. ACS ES T Water.

[40] Kaczmarek H., Kowalonek J., Ołdak D. (2003). The influence of UV-irradiation on poly(vinyl chloride) modified by iron and cobalt chlorides. Polym. Degrad. Stab..

[41] van der Ven L., van Gemert M., Batenburg L., Keern J., Gielgens L., Koster T., Fischer H. (2000). On the action of hydrotalcite-like clay materials as stabilizers in polyvinylchloride. Appl. Clay Sci..

[42] Lin Y.-J., Li D.-Q., Evans D.G., Duan X. (2005). Modulating effect of Mg–Al–CO_3_ layered double hydroxides on the thermal stability of PVC resin. Polym. Degrad. Stab..

