# Supplementary material for: Analysis of Additive Distributions in Rigid PVC by Laser Ablation Inductively Coupled Plasma Mass Spectrometry
Source: Polymers (Basel). 2026 Oct 23;18(21):2626. doi: 10.3390/polym18212626 (PMC13765876; doi:10.3390/polym18212626)
Supplement: Supplementary file 1 [file polymers-18-02626-s001.zip › polymers-4627702-supplementary.pdf]

polymers

MDPI

Supplementary Material

Analysis of Additive Distributions in Rigid PVC by Laser Ablation Inductively Coupled Plasma Mass Spectrometry

Saskia Kerkeling [1,*], Martin Kreyenschmidt [1], Christian Gieler [2], Matthias Kaltefleiter-Jürgens [2], Michel Sieffert [3] and Ursula E. A. Fittschen [4]

[1] Department of Chemical Engineering, FH Münster University of Applied Sciences, 48565 Steinfurt, Germany
[2] VEKA AG, 48324 Sendenhorst, Germany; cgieler@veka.com (C.G.)
[3] Gealan Fenster-Systeme GmbH, 95145 Oberkotzau, Germany; msieffert@gealan.de
[4] Institute of Inorganic and Analytical Chemistry, Clausthal University of Technology, 38678 Clausthal, Germany; ursula.fittschen@tu-clausthal.de
* Correspondence: saskia.Kerkeling@fh-muenster.de

Dry-blend Preparation

Table S1 Raw materials used for the dry-blend production

Raw Material	Content (wt%)	Description	Manufacturer
PVC	83	K-value: 65	VESTOLIT, Germany
Impact modifier	1.6	Acrylic polymer with core-shell structure	Dow, Germany
Processing Aid	0.4	Acrylic processing aid	Röhm, Germany
Internal lubricant	0.07	Ethylene-vinyl acetate copolymer wax	Innospec, Germany
External lubricant	0.13	Paraffin wax	Akdeniz Chemson, Germany
Chalk	9.6	Purity: 99.3%	Omya, Switzerland
TiO_2	2.8	Purity: ≥ 92.5%	Kronos, Germany
Stabilizer One Pack	2.4	Ca/Zn stabilizer system	IKA, Germany

The PVC dry-blend was prepared in a 1100 L vertical high-speed mixer (model 0359/M1, MTI, Germany) and subsequently cooled in a 3590 L horizontal cooling mixer (model 976, MTI, Germany).

Pressed Sheets

Table S2 Two-roller mill LRM-SC-110/T3E (Labtech Engineering, Thailand) operating conditions

Variable	
Roller temperatures front/rear (°C)	168/160
Rotational speed front/rear (rpm)	36/30
Slit width (mm)	0.11[a]/0.3
Rolling time (min)	2

[a] for the first 30 seconds

Table S3 Hydraulic press LP-S20 (Labtech Engineering, Thailand) operating conditions

Variable	
Temperature (°C)	175
Contact pressure (kN)	75–80
Holding time (s)	90
Cooling time (s)	30

Extruded Belts

Extruded belts were processed using HAAKE™ PolyLab OS Rheodrive 7 with HAAKE™ Rheomex OS single-screw extruder, sheet tool, and three-roll sheet take-off (Thermo Fisher Scientific, Germany).

Table S4 Single-screw extruder operating conditions

Variable			Duration (min)
Extruder speed (rpm)	Step 1	40	5
	Step 2	60	4
Temperature (°C)	Zone 1	165	
	Zone 2	167	
	Zone 3	170	

Window Profile

Table S5 Process parameters of extrusion for window profiles

Variable	Co-extruded Profile	Mono-extruded Profile
Extruder model	Main extruder: DS11.26<br>Co-extruder: CE 7 Z	KMD 112.5–25
Extruder type	Conical	Parallel
Extruder manufacturer	Hans WEBER Maschinenfabrik (Germany)	KraussMaffei Group GmbH (Germany)
Melt temperature (°C)	Main extruder: 194<br>Co-extruder: 191	192
Melt pressure (bar)	Main extruder: 326<br>Co-extruder: 448	387
Extruder speed (rpm)	Main extruder: 14.7<br>Co-extruder: 15.1	14.7

Color Shift after Artificial Weathering of Window Profiles

Table S6 Color shift in the CIELAB color space after 3000 h Q-SUN weathering

Coordinates	Color Shift
ΔL*	0.76
Δa*	-0.27
Δb*	-1.67
ΔE*	1.85

https://doi.org/10.3390/xxxxx
